# Use of Antihyperglycemic Medications Among US People with Limited English Proficiency

**DOI:** 10.1007/s11606-025-09385-x

**Published:** 2025-01-28

**Authors:** Frank Müller, Harland Holman, Nikita Bhangu, Jepkoech Kottutt, Hend Azhary, Omayma Alshaarawy

**Affiliations:** 1 Department of Family Medicine, College of Human Medicine, Michigan State University, Grand Rapids, MI 49503 USA; 2 Spectrum Health Family Medicine Residency Clinic, Grand Rapids, MI 49503 USA; 3 Department of General Practice, University Medical Center Göttingen, Humboldtallee 38, 37073 Göttingen, Germany; 4 Department of Family Medicine, College of Human Medicine, Michigan State University, East Lansing, MI 48824 USA

**Keywords:** limited English proficiency, migrants, medication adherence, HbA1c, diabetes mellitus, NHANES

## Abstract

**Background:**

Language barriers can impact pharmaceutical disease management leading to potential health disparities among limited English proficiency (LEP) people with diabetes mellitus (DM) in the United States (US).

**Objective:**

To assess the use of antihyperglycemic medications and estimate their impact on glycemic control by LEP status.

**Design:**

Cross-sectional design. We compared the classes of prescribed antihyperglycemic medications and their impact on glycemic control between English-speaking and LEP participants (i.e., Spanish-speaking or needing interpretation services) with DM applying generalized linear models and adjusting for sociodemographic variables.

**Participants:**

Data from the US National Health and Nutrition Examination Survey (NHANES 2003–2018).

**Main Measures:**

Selected language for interview or interpreter request (main exposure). Outcomes include prescribed antihyperglycemic medications and glycemic control (HBA1c).

**Key Results:**

Data for 4666 participants with DM were analyzed. Antihyperglycemic medications were similarly used by LEP and English-speaking people with DM, except for insulin, which was less frequently used by LEP people. Despite similar medications, LEP people using biguanides and TZDs were less likely to reach glycemic target levels (adjusted odds ratios ranging 1.7 to 3.3) compared to English-speaking people with DM.

**Conclusions:**

Our findings indicate that the differences in DM outcomes among LEP people are likely attributed to factors other than medication prescription. These might include cultural beliefs, dietary adjustments, and communication barriers in healthcare. Enhanced patient education, acknowledgment of cultural practices, and improved language services could potentially mitigate these disparities.

## INTRODUCTION

The latest census reports that 22% of United States (US) residents speak a language other than English at home and approximately 20% of these residents reported speaking English “not well” or “not at all”.^1^ People with LEP often experience challenges accessing and navigating the healthcare system and are more prone to unfavorable outcomes of chronic conditions and medical errors compared to English-speaking people.^2,3^ This also applies to the management of diabetes mellitus (DM): people with LEP are more likely to have undiagnosed DM,^4^ and struggle more often to maintain blood glucose levels within target range^5–7^ and cardiovascular health goals compared to English-speaking people.^8^ The reason for these findings is complex: language discordance may impede DM self-management,^9,10^ provider’s lack of cultural competency in DM care, e.g., lacking awareness on dietary habits,^11^ or religious and cultural beliefs.^12^ Migrant patients also have expressed anxiety about encounters with native English-speaking providers, despite the availability of language interpretation services.^13^

Furthermore, previous studies have highlighted variations in medication prescribing patterns for DM by race and ethnicity^14^ and other studies have suggested that people from racial and ethnic minority groups are likely to experience decreased initiation of newer antihyperglycemic medications and technologies with high effectiveness and cardiovascular benefits for patients with DM.^15–17^ In addition to socioeconomic factors, differences in medication access might be attributed to language discordance and limited opportunities for effective counseling on medication regimens. Indeed, adequate comprehension of medication dosing, timing, and side effects is required to minimize the incidence of DM complications, including life-threatening hyperglycemia.^18^ Healthcare providers might prioritize medications with simple regimens to minimize these risks. To date, the relationship between LEP and the pharmacologic management of DM has not been assessed in a representative sample of the US population. Therefore, this study used data from the National Health and Nutrition Examination Survey (NHANES) to evaluate the use of antihyperglycemic medications and estimate their impact on glycemic control by LEP status.

## METHODS

### Study Design

The NHANES is designed to enroll and assess a representative sample of approximately 5000 participants/year of the US civilian non-institutionalized population. Participants are selected using a multistage area probability sampling approach^19^ with certain racial and ethnic groups being deliberately oversampled to enhance validity. Data were derived from computer-assisted interviews, physical exams, and laboratory tests. For this study, we used data from eight NHANES cycles (2003–2004, 2005–2006, 2007–2008, 2009–2010, 2011–2012, 2013–2014, 2015–2016, 2017–2018).

### Study Subjects

We used data for non-pregnant adults (aged 18 or older) with diagnosed DM (i.e., answered “yes” to the question “Other than during pregnancy, have you ever been told by a doctor or health professional that you have diabetes or sugar diabetes?”; *n* = 5467). Participants were excluded from the analysis if they were solely using insulin or received their DM diagnosis before the age of 18 as both aspects are predominately prevalent among patients with type 1 DM (*n* = 797). We further excluded four participants who solely used less commonly prescribed medications (amylin analog or alpha glucosidase).

### Main Exposure

In NHANES, participants selected the language of the interview (English or Spanish) or requested an interpreter to complete the interviews. We categorized the main exposure for this study as (a) interview completed in English (i.e., English-speaking), (b) interview completed in Spanish (i.e., LEP-Spanish), or (c) interview completed with an interpreter (i.e., LEP-interpreter).

### Outcome

Here, the outcomes of interest were classes of antihyperglycemic medication and glycemic control. The use of prescribed medications was assessed during the NHANES household interview. Participants who responded “yes” to the question “In the past 30 days, have you used or taken medication for which a prescription is needed?” were asked to show the interviewer the medication container of all the products used or verbally report the name of the medication(s). Antihyperglycemic medications were categorized into biguanides (i.e., metformin), insulin secretagogues (i.e., sulfonylurea, meglitinide), thiazolidinediones (TZDs), antidiabetic combinations (two or more classes of antidiabetic medications), and a group with newer medications including glucagon-like peptide 1 agonists (GLP1), dipeptidyl peptidase 4 inhibitors (DDP4), and sodium-glucose cotransporter-2 inhibitors (SGLT2). A final group included any of the beforementioned medication(s) plus insulin. The categorization followed the therapeutic drug classes provided by NHANES, with insulin secretagogues and newer antidiabetic drugs being combined into respective groups due to small sample sizes.

Glycemic control was evaluated using HbA1c that reflects average blood glucose levels for the preceding 90 days. The threshold for above-target glycemic levels was defined as HbA1c > 7% (> 53 mmol/mol) as defined in the current guidelines from American Diabetes Association.^20^

### Study Covariates

Additional covariates included participants’ survey reported age (years), sex (male/female), educational attainment (high school degree or less/higher than high school degree), ratio of family income to federal poverty guidelines (< 1/1 + /missing), health insurance coverage (any type of healthcare plan; yes/no), time since DM diagnosis (years), number of healthcare visits in the past 12 months (≤ 1/ > 1), number of prescribed antihyperglycemic medications (range 0–5), and body mass index (BMI, measured by study personnel as part of the NHANES physical exam and categorized into non-obese (< 30 kg/m^2^), obese (≥ 30 kg/m^2^) or missing).

### Statistical Analysis

First, we used descriptive statistics to characterize the study sample by LEP status, including relative and absolute frequencies, mean, and standard error of mean (SEM). Differences between LEP status were assessed using analysis of variance for continuous variables and design-corrected Rao-Scott *x*^2^ test for categorical variables. The crude prevalence of antihyperglycemic medication use was estimated for each LEP group. Separate logistic regression models were then used to estimate the adjusted prevalence of each medication use by LEP status. Multivariable logistic regression was also used to estimate the odds of above-target glycemic levels by LEP status, stratified by antihyperglycemic medication use.

All analyses were adjusted for the NHANES complex sampling design (clustering, stratification, data release cycle, and NHANES examination weights). Data analyses were conducted with SAS® version 9.4 (SAS Institute, Inc., Cary, NC). The significance level was set, a priori, at *p* < 0.05.

### Research Ethics

NHANES received approval through the National Center for Health Statistics Research Ethics Review Board.^21^ All participants provided written informed consent prior to enrollment. The Michigan State University Institutional Review Board deemed the analysis of the publicly available deidentified NHANES data as non-human subject research.

## RESULTS

This study included 4666 participants with self-reported physician-diagnosed probable type 2 DM (age of diagnosis ≥ 18 years; not solely using insulin). Most participants completed the NHANES interview in English (90.0%), whereas 7.0% of the participants completed the interview in Spanish and 3.0% requested an interpreter. Comparing the three groups (Table 1), age, antihyperglycemic medication use (yes/no), education, health insurance, healthcare visits, income to poverty ratio, BMI, and HbA1c levels were statistically different. For example, LEP-Spanish (85.8%) and LEP-interpreter (81.3%) participants more commonly held a high school degree or less as their highest level of educational attainment compared to English-speaking participants (46.5%). Also, LEP-Spanish (36.8%) and LEP-interpreter (19.5%) participants were more likely to lack health insurance coverage than English-speaking participants (8.0%).

**Table 1:** Selected Characteristics of the Study Population. Data Are for the US NHANES, 2003–2018

**LEP status**	**English-speaking** **(*n* = 3801)**	**LEP-Spanish** **(*n* = 665)**	**LEP-interpreter** **(*n* = 200)**	
**Characteristics**	Mean (SEM) or unweighted *n* (weighted %)			*p*-value*
**Age, years**	60.8 (0.3)	56.1 (0.7)	63.3 (1.2)	< 0.001
**Time since DM diagnosis, years**	9.6 (0.2)	9.4 (0.4)	8.8 (0.6)	0.46
**Prescription antihyperglycemic medications**^**†**^				
Not using	767 (20.3%)	159 (26.6%)	32 (15.0%)	0.004
Mean number if using	1.7 (0.02)	1.6 (0.04)	1.5 (0.06)	0.10
**Female sex**	1839 (49.4%)	340 (52.3%)	100 (55.9%)	0.13
**High school degree or less**	2003 (46.5%)	591 (85.8%)	160 (81.3%)	< 0.001
**Do not have health insurance coverage**	340 (8.0%)	205 (36.9%)	46 (19.5%)	< 0.001
**Annual healthcare visits > 1**	3489 (92.0%)	544 (78.0%)	178 (90.2%)	< 0.001
**Family income to poverty ratio**				< 0.001
< 1	653 (12.3%)	264 (39.2%)	104 (53.3%)	
≥ 1	2815 (80.1%)	285 (45.5%)	67 (35.1%)	
Missing	333 (7.6%)	116 (15.3%)	29 (11.6%)	
**HbA1c**				< 0.001
≤ 7%	2105 (57.4%)	296 (44.0%)	88 (46.9%)	
> 7%	1516 (39.1%)	347 (53.0%)	98 (46.9%)	
Missing	180 (3.5%)	22 (3.0%)	14 (6.2%)	
**BMI**				< 0.001
< 30 kg/m^2^	1525 (35.5%)	322 (48.4%)	142 (69.5%)	
≥ 30 kg/m^2^	2187 (62.5%)	325 (49.3%)	55 (26.8%)	
Missing	89 (2.0%)	18 (2.3%)	3 (3.7%)	

^*^p-values are based on analysis of variance for continuous variables and design-corrected Rao-Scott *x*^2^ test for categorical variables

^**†**^Prescription antihyperglycemic medication use was defined as taking biguanides, insulin secretagogues, TZDs, antihyperglycemic combinations, GLP1 agonists, DDP4 inhibitors, or SGLT2 inhibitors in the 30 days prior to NHANES assessment, with or without insulin

**Figure 1:**
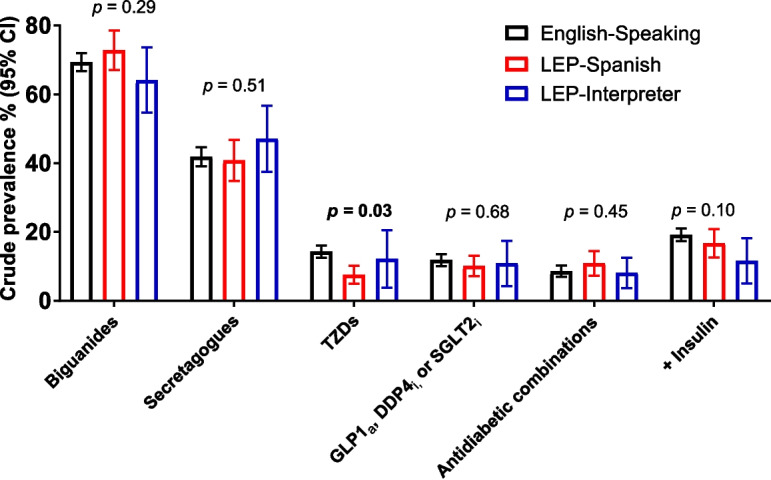
Use of antihyperglycemic medications by LEP status among participants who used at least one medication in the past 30 days (*n* = 3708). Data are for the US NHANES, 2003–2018.

Figure 1 presents the crude prevalence of antihyperglycemic medication use by LEP status among participants who used at least one medication in the past 30 days. The use of biguanides, secretagogues, antihyperglycemic combinations, or newer medications (GLP1 agonists, DDP4 inhibitors, or SGLT2 inhibitors), with or without insulin, did not differ by LEP status (all *p* > 0.05). The use of TZDs was different across the three groups (*p* = 0.03), being less commonly used by LEP-Spanish participants with DM.

When adjusting for potentially confounding variables, the association between LEP-Spanish and the use of any antihyperglycemic medication was attenuated towards the null (odds ratio (OR) = 1.0; 95% CI = 0.8, 1.4), whereas participants in the LEP-interpreter group were more likely to use antihyperglycemic medications compared to English-speaking participants (OR = 1.8; 95% CI = 1.1, 2.9), with income and health insurance status explaining the changes from the unadjusted estimates (Table 2, panel A). Among participants reporting to have taken antihyperglycemic medications in the past 30 days, LEP status was not associated with medication use without insulin (Table 2, panel B). On the other hand, medication use with insulin was less common among LEP-Spanish (OR = 0.6; 95% CI = 0.4, 0.9) and LEP-interpreter participants (OR = 0.5; 95% CI = 0.3, 1.0) compared to English-speaking participants, but these differences were not statistically significant for LEP-interpreter participants (*p* = 0.07).

**Table 2:** Association Between LEP Status and Antihyperglycemic Medication Use. Data Are for the US NHANES, 2003–2018

LEP status	English-speaking	LEP-Spanish	LEP-interpreter
	**Odds ratios of use (95% CI)**		
**Panel A**: All participants (*n* = 4666)			
**Any antihyperglycemic medication**			
Model1^a^	1 (reference)	**0.7 (0.5, 0.9)**	1.4 (0.9, 2.2)
Model 2^b^	1 (reference)	**0.8 (0.6, 0.9)**	1.4 (0.9, 2.2)
Model 3^c^	1 (reference)	1.0 (0.8, 1.4)	**1.8 (1.1, 2.9)**
**Panel B**: Participants who used any antihyperglycemic medication in the past 30 days (*n* = 3708)			
**Biguanides**			
Model 1^a^	1 (reference)	1.1 (0.8, 1.5)	0.7 (0.5, 1.1)
Model 2^b^	1 (reference)	1.0 (0.7, 1.4)	0.8 (0.5, 1.2)
Model 4^d^	1 (reference)	1.1 (0.8, 1.6)	0.7 (0.5, 1.2)
**Insulin secretagogues**			
Model 1^a^	1 (reference)	1.0 (0.8, 1.3)	1.3 (0.9, 1.9)
Model 2^b^	1 (reference)	1.1 (0.8, 1.5)	1.3 (0.9, 1.8)
Model 4^d^	1 (reference)	1.1 (0.8, 1.6)	1.2 (0.8, 1.9)
**TZDs**			
Model 1^a^	1 (reference)	**0.6 (0.4, 0.9)**	0.9 (0.4, 2.0)
Model 2^b^	1 (reference)	**0.6 (0.4, 0.9)**	1.0 (0.4, 2.1)
Model 4^d^	1 (reference)	0.6 (0.4, 1.0)	1.2 (0.5, 3.1)
**Newer medications**			
Model 1^a^	1 (reference)	0.8 (0.6, 1.1)	0.8 (0.4, 1.5)
Model 2^b^	1 (reference)	0.8 (0.5, 1.1)	0.8 (0.5, 1.6)
Model 4^d^	1 (reference)	0.8 (0.5, 1.2)	1.0 (0.4, 2.3)
**Antidiabetic combination**			
Model 1^a^	1 (reference)	1.3 (0.8, 2.1)	1.0 (0.5, 1.8)
Model 2^b^	1 (reference)	1.3 (0.8, 2.1)	1.0 (0.6, 1.9)
Model 4^d^	1 (reference)	1.2 (0.8, 1.9)	1.0 (0.5, 1.9)
** + Insulin**			
Model 1^a^	1 (reference)	0.8 (0.6, 1.1)	0.5 (0.3, 1.0)
Model 2^b^	1 (reference)	0.8 (0.6, 1.1)	0.5 (0.3, 1.0)
Model 4^d^	1 (reference)	**0.6 (0.4, 0.9)**	0.5 (0.3, 1.0)

^a^Logistic regression models are adjusted for NHANES survey design including NHANES data cycle

^b^Logistic regression models are additionally adjusted for participant’s age (years) and sex (male, female)

^c^Logistic regression models are additionally adjusted for time since diabetes diagnosis (years), education attainment (high school or less, > high school), income to poverty ratio (< 1, 1 + , missing), health insurance coverage (yes, no), number of healthcare visits in the past 12 months, BMI (< 30 kg/m^2^, 30 + kg/m^2^, missing), and HbA1c ≤ 7%, > 7%, missing)

^d^Logistic regression models are additionally adjusted for the number of antihyperglycemic medications used

We then ran a logistic regression model to estimate the association between above-target glycemic HbA1c (> 7%) and LEP status, adjusting for potential confounding variables and medication use. Overall, favorable HbA1c targets control were less often reached by LEP-Spanish and LEP-interpreter participants compared to English-speaking participants (Table 3). To further evaluate these differences, we ran stratified analysis by type of medication. In fully adjusted models, LEP people using biguanides showed higher odds of elevated HbA1c (LEP Spanish OR = 1.8; 95% CI = 1.2, 2.5 and LEP interpreter OR = 1.9; 95% CI = 1.1, 3.1) compared to English-Speaking participants. Among TZD users, this association was significant only for Spanish-speaking LEP people (OR = 3.5; 95% CI = 1.7, 7.2), while among insulin secretagogue users, it was significant only for interpreter-LEP people (OR = 2.0; 95% CI = 1.04, 3.8). No significant associations were observed among participants using newer medications or antidiabetic combinations.

**Table 3:** Association Between Above Target HbA1c (i.e., HbA1c > 7%) and LEP Status, Stratified by Prescription Antihyperglycemic Medication Use. Data Are for the US NHANES, 2003–2018

LEP status	English-speaking	LEP-Spanish	LEP-interpreter
	**Odds ratios of above-target HbA1c (95% CI)**		
Participants who used any antihyperglycemic medication (*n* = 3708)			
Model 1^a^	1 (reference)	1.8 (1.4, 2.3)	1.5 (0.9, 2.4)
Model 2^b^	1 (reference)	1.7 (1.3, 2.2)	1.6 (1.02, 2.6)
Model 3^c^	1 (reference)	1.6 (1.2, 2.1)	1.8 (1.1, 2.8)
Participants who used biguanides (*n* = 2510)			
Model 1^a^	1 (reference)	2.0 (1.4, 2.6)	1.5 (0.9, 2.4)
Model 2^b^	1 (reference)	1.9 (1.4, 2.5)	1.6 (1.01, 2.6)
Model 4^d^	1 (reference)	1.8 (1.2, 2.5)	1.9 (1.1, 3.1)
Participants who used insulin secretagogues (*n* = 1623)			
Model 1^a^	1 (reference)	2.0 (1.3, 2.9)	1.7 (0.8, 3.5)
Model 2^b^	1 (reference)	1.7 (1.1, 2.6)	1.9 (1.0, 3.6)
Model 4^d^	1 (reference)	1.4 (0.9, 2.1)	2.0 (1.04, 3.8)
Participants who used TZDs (*n* = 474)			
Model 1^a^	1 (reference)	3.2 (1.6, 6.6)	2.1 (0.4, 12.0)
Model 2^b^	1 (reference)	3.3 (1.7, 6.6)	2.1 (0.4, 12.9)
Model 4^d^	1 (reference)	3.5 (1.7, 7.2)	3.3 (0.9, 12.4)
Participants who used newer medications (*n* = 386)			
Model 1^a^	1 (reference)	1.4 (0.7, 3.2)	1.4 (0.4, 5.2)
Model 2^b^	1 (reference)	1.4 (0.6, 3.2)	1.3 (0.4, 4.7)
Model 4^d^	1 (reference)	1.1 (0.4, 2.6)	1.1 (0.3, 3.5)
Participants who used antidiabetic combination (*n* = 333)			
Model 1^a^	1 (reference)	0.9 (0.4, 1.8)	1.1 (0.3, 3.8)
Model 2^b^	1 (reference)	0.9 (0.4, 2.1)	1.2 (0.3, 4.4)
Model 4^d^	1 (reference)	1.0 (0.4, 2.5)	1.3 (0.3, 5.5)
Participants who used any antihyperglycemic medication and insulin (*n* = 673)			
Model 1^a^	1 (reference)	2.1 (1.1, 4.1)	3.5 (0.4, 29.2)
Model 2^b^	1 (reference)	1.9 (1.0, 3.8)	3.2 (0.4, 24.6)
Model 4^d^	1 (reference)	1.8 (0.8, 3.7)	3.3 (0.4, 25.0)

^a^Logistic regression models are adjusted for NHANES survey design including NHANES data cycle

^b^Logistic regression models are additionally adjusted for age (years) and sex (male, female)

^c^Logistic regression models are additionally adjusted for time since diabetes diagnosis (years), education attainment (high school or less, > high school), income to poverty ratio (< 1, 1 + , missing), health insurance coverage (yes, no), number of healthcare visits in the past 12 months, BMI (< 30 kg/m^2^, 30 + kg/m^2^, missing), antihyperglycemic medication (biguanides, insulin secretagogues, TZDs, newer medications, and antidiabetic combination), the number of antihyperglycemic medication used, and insulin use (yes, no)

^d^Logistic regression models are additionally adjusted for time since diabetes diagnosis (years), education attainment (high school or less, > high school), income to poverty ratio (< 1, 1 + , missing), health insurance coverage (yes, no), number of healthcare visits in the past 12 months, BMI (< 30 kg/m^2^, 30 + kg/m^2^, missing), the number of antihyperglycemic medication used, and insulin use (yes, no; except when specified as the outcome)

## DISCUSSION

Our study on self-reported medication use in NHANES data from 2003 to 2018 has two main findings: First, antidiabetic medication does not significantly differ between English-speaking and LEP (both Spanish-speaking or other LEP using an interpreter) people with DM whereas the prevalence of insulin use was lower among LEP-Spanish people compared to English-speaking people. Second, despite widely similar treatment regimes in LEP and English-speaking people, LEP people were less likely to reach glycemic target levels compared to English-speaking people. The differences in glycemic control by LEP status were attenuated when using newer medications or antidiabetic combinations.

While there is a growing body of evidence suggesting that DM outcomes in language-diverse people are worse compared to English speakers,^5–7,22^ this study suggests that these differences are unlikely due to differences in the prescription of antihyperglycemic medications. Our findings point to systemic barriers in healthcare delivery, including structural obstacles to accessing care, provider-level factors such as medication inertia, and challenges in providing culturally appropriate care.^23^ Furthermore, DM management require lifestyle adjustments, including diet, exercise, reduction of alcohol use, and smoking cessation as well as stress management.^24–27^ Research has highlighted how health system barriers and limited access to appropriate resources can affect medication use patterns and lifestyle modifications among migrant populations with DM.^7,28^

Achieving diet and drug adherence also requires effective and empathetic communication strategies between providers and their patients, despite language discordance. The utilization of professional interpreters in the provision of DM care to LEP populations has been associated with improved glycemic control.^29^ However, studies with LEP patients in hospital settings have confirmed that relevant information or treatment considerations are often provided sparsely, e.g., by underutilization of teach-back methods or information not provided in the primary spoken language.^30^ Providers often find the utilization of interpreters time-consuming and feel uncomfortable relying on them,^31^ leading to underutilization and “getting-by” with untrained ad hoc interpreters^32,33^ resulting in suboptimal care.

Providing DM care in patients’ native languages has also been shown to improve outcomes,^34^ but multilingual healthcare providers in the US and other Western countries are sparse^35,36^ and most US medical schools do not offer language courses in which future provider could acquire language competencies in another language than English.^37^

Besides physicians, pharmacists play a crucial role in improving medication adherence and reducing adverse effects by providing essential education on prescribed medication.^38–40^ However, language discordance makes it less likely that LEP people receive this education, and several studies have underscored the lack of interpretation capacities in pharmacies.^41–44^

Some LEP and immigrant populations might perceive DM not as a medical condition but as a moral shortcoming. These beliefs can obstruct lifestyle changes as well as disease self-management and healthcare providers who lack knowledge about cultural beliefs and practices may impede effective glycemic control. A study of DM perceptions among Chinese Americans found that the diagnosis was perceived as a punishment for actions performed in a past life.^45^ Traditional Chinese medicine focuses on the body’s harmony and its environment rather than a scientific and biomedical disease perception^46,47^ and thus may contribute to delayed treatment for the rather asymptomatic DM condition. Adopting Western medical treatments may cause emotional stress for patients and reluctance in drug adherence due to the incongruence with their cultural belief system. Another study has shown that Spanish-dominant and bilingual Latinos were significantly more likely than their English-dominant counterparts to believe that diet, exercise, and weight would have only a little or no impact on their health.^48^ These illustrative examples, mainly based on qualitative and ethnographic studies,^49^ demonstrate possible cultural perspectives without indicating how common such beliefs may be. While these studies cannot be generalized across diverse immigrant populations, they should remind providers to remain attentive to individual cultural contexts in diabetes care.

Besides cultural perceptions of DM, new migrants to the US, including those with DM, face challenges in retaining the diet that they were used to in their countries of origin.^50^ Contributing factors that make migrants switch to a Western diet (“dietary acculturation”) and thus more unfavorable for glycemic control^51^ include language barriers, limited financial resources, lack of mobility, and unavailability of suitable grocery stores (“food deserts”).^52^ Additionally, as migrants are often underinsured,^53,54^ glucose monitoring as part of their DM management can be challenging and thus impeding glycemic control.^55^

Our study comes with several limitations. While we excluded participants who received DM diagnosis before the age of 18 and participants who solely use insulin, we cannot ascertain the type of DM. This study is based on NHANES data spanning from 2003 to 2018 and may not represent recent changes in DM trends among LEP populations, e.g., recent FDA approvals or warnings related to antihyperglycemic medications. The NHANES program was suspended in 2020 due to the COVID-19 pandemic, and the NHANES 2019–2020 data are not nationally representative, and no other data has been released since the pandemic. Furthermore, we used a pragmatic way to define LEP through interpretation service utilization for the respective NHANES interviews and thus cannot provide information on the proficiency level of the English language or provide stratified analyses on different language subgroups other than Spanish. Additionally, we were not able to assess if NHANES participants had access to language-concordant providers. While a strength of our study is the use of a large representative sample, NHANES is unlikely to have captured the large number of seasonal migrant workers in the US.

Despite these limitations, specific treatment formats in respective languages that focus on enhancing DM self-management and lifestyle changes, while actively considering cultural beliefs and practices, could lead to better DM outcomes among people with LEP.

## Data Availability

All data analyzed for this manuscript are publicly available through the NHANES Study website (https://www.cdc.gov/nchs/nhanes/).

## References

[1] **Dietrich S, Hernandez E.** Language Use in the United States: 2019. American Community Survey Reports. Washington D.C.: United States Census Bureau 2022 [cited 21 Dec 2024]. Available from: https://www.census.gov/content/dam/Census/library/publications/2022/acs/acs-50.pdf.

[2] **Karliner LS, Kim SE, Meltzer DO, Auerbach AD.** Influence of language barriers on outcomes of hospital care for general medicine inpatients. J Hosp Med. 2010; 5:276–82. 10.1002/jhm.658 PMID: 20533573 . 20533573 10.1002/jhm.658

[3] **Twersky SE, Jefferson R, Garcia-Ortiz L, Williams E, Pina C.** The Impact of Limited English Proficiency on Healthcare Access and Outcomes in the U.S.: A Scoping Review. Healthcare (Basel). 2024; 12. 10.3390/healthcare12030364. 10.3390/healthcare12030364 PMC10855368 38338249

[4] **Holman H, Müller F, Bhangu N, Kottutt J, Alshaarawy O.** Impact of Limited English Proficiency on the Diagnosis and Awareness of Diabetes: The National Health and Nutrition Examination Survey, 2003–2018. Diabetes Care. 2022. 10.2337/dc22-0594. 10.2337/dc22-0594 PMC9346981 35763452

[5] **Fernandez A, Schillinger D, Warton EM, Adler N, Moffet HH, Schenker Y, et al.** Language barriers, physician-patient language concordance, and glycemic control among insured Latinos with diabetes: the Diabetes Study of Northern California (DISTANCE). J Gen Intern Med. 2011; 26:170–6. Epub 2010/09/29. 10.1007/s11606-010-1507-6 PMID: 20878497 . 20878497 10.1007/s11606-010-1507-6 PMC3019330

[6] **Mehler PS, Lundgren RA, Pines I, Doll K.** A community study of language concordance in Russian patients with diabetes. Ethn Dis. 2004; 14:584–8. 15724780

[7] **Traylor AH, Schmittdiel JA, Uratsu CS, Mangione CM, Subramanian U.** Adherence to cardiovascular disease medications: does patient-provider race/ethnicity and language concordance matter. J Gen Intern Med. 2010; 25:1172–7. Epub 2010/06/23. 10.1007/s11606-010-1424-8 PMID: 20571929 . 20571929 10.1007/s11606-010-1424-8 PMC2947630

[8] **Holman H, Müller F, Bhangu N, Kottutt J, Alshaarawy O.** Impact of limited English proficiency on the control of diabetes and associated cardiovascular risk factors. The National Health and Nutrition Examination Survey, 2003-2018. Prev Med. 2023; 167:107394. Epub 2022/12/20. 10.1016/j.ypmed.2022.107394 PMID: 36563970 . 36563970 10.1016/j.ypmed.2022.107394

[9] **Wilson E, Chen AHM, Grumbach K, Wang F, Fernandez A.** Effects of limited English proficiency and physician language on health care comprehension. J Gen Intern Med. 2005; 20:800–6. 10.1111/j.1525-1497.2005.0174.x PMID: 16117746 . 16117746 10.1111/j.1525-1497.2005.0174.x PMC1490205

[10] **Campos C.** Addressing cultural barriers to the successful use of insulin in Hispanics with type 2 diabetes. South Med J. 2007; 100:812–20. 10.1097/SMJ.0b013e3180f609c4 PMID: 17713308 . 17713308 10.1097/SMJ.0b013e3180f609c4

[11] **Patel NR, Kennedy A, Blickem C, Reeves D, Chew-Graham C.** "I'm Managing My Diabetes between Two Worlds": Beliefs and Experiences of Diabetes Management in British South Asians on Holiday in the East--A Qualitative Study. J Diabetes Res. 2016; 2016:5436174. Epub 2015/11/30. 10.1155/2016/5436174 PMID: 26697499 . 26697499 10.1155/2016/5436174 PMC4677201

[12] **Moreira T, Hernandez DC, Scott CW, Murillo R, Vaughan EM, Johnston CA.** Susto, Coraje, y Fatalismo: Cultural-Bound Beliefs and the Treatment of Diabetes Among Socioeconomically Disadvantaged Hispanics. Am J Lifestyle Med. 2018; 12:30–3. Epub 2017/11/02. 10.1177/1559827617736506 PMID: 29541008 . 29541008 10.1177/1559827617736506 PMC5847327

[13] **Choi SE, Lee JJ, Park JJ, Sarkisian CA.** Spousal support in diabetes self-management among Korean immigrant older adults. Res Gerontol Nurs. 2015; 8:94–104. Epub 2014/12/14. 10.3928/19404921-20141120-01 PMID: 25420183 . 25420183 10.3928/19404921-20141120-01 PMC4443801

[14] **Lamprea-Montealegre JA, Madden E, Tummalapalli SL, Peralta C, Neilands TB, Garcia PK, et al.** Association of Race and Ethnicity With Prescription of SGLT2 Inhibitors and GLP1 Receptor Agonists Among Patients With Type 2 Diabetes in the Veterans Health Administration System. JAMA. 2022; 328:861–71. 10.1001/jama.2022.13885 PMID: 36066519 . 36066519 10.1001/jama.2022.13885 PMC9449794

[15] **Elhussein A, Anderson A, Bancks MP, Coday M, Knowler WC, Peters A, et al.** Racial/ethnic and socioeconomic disparities in the use of newer diabetes medications in the Look AHEAD study. Lancet Reg Health Am. 2022; 6. 10.1016/j.lana.2021.100111. 10.1016/j.lana.2021.100111 PMC8920048 35291207

[16] **Mathur R, Farmer RE, Eastwood SV, Chaturvedi N, Douglas I, Smeeth L.** Ethnic disparities in initiation and intensification of diabetes treatment in adults with type 2 diabetes in the UK, 1990-2017: A cohort study. PLoS Med. 2020; 17:e1003106. Epub 2020/05/15. 10.1371/journal.pmed.1003106 PMID: 32413037 . 32413037 10.1371/journal.pmed.1003106 PMC7228040

[17] **O'Connor MR, Carlin K, Coker T, Zierler B, Pihoker C.** Disparities in Insulin Pump Therapy Persist in Youth With Type 1 Diabetes Despite Rising Overall Pump Use Rates. J Pediatr Nurs. 2019; 44:16–21. Epub 2018/10/16. 10.1016/j.pedn.2018.10.005 PMID: 30581163 . 30581163 10.1016/j.pedn.2018.10.005 PMC10602396

[18] **Sarkar U, Karter AJ, Liu JY, Moffet HH, Adler NE, Schillinger D.** Hypoglycemia is more common among type 2 diabetes patients with limited health literacy: the Diabetes Study of Northern California (DISTANCE). J Gen Intern Med. 2010; 25:962–8. Epub 2010/05/18. 10.1007/s11606-010-1389-7. PMID: 20480249 . 20480249 10.1007/s11606-010-1389-7 PMC2917655

[19] National Center for Health Statistics. NHANES Survey Methods and Analytic Guidelines [updated 8 Feb 2022; cited 8 Feb 2022]. Available from: https://wwwn.cdc.gov/nchs/nhanes/AnalyticGuidelines.aspx.

[20] **Draznin B, Aroda VR, Bakris G, Benson G, Brown FM, Freeman R, et al.** 10. Cardiovascular Disease and Risk Management: Standards of Medical Care in Diabetes-2022. Diabetes Care. 2022; 45:S144-S174. 10.2337/dc22-S010 PMID: 34964815 . 34964815 10.2337/dc22-S010

[21] National Center for Health Statistics. NHANES - Research Ethics Review Board Approval [updated 9 Feb 2022; cited 9 Feb 2022]. Available from: https://www.cdc.gov/nchs/nhanes/irba98.htm.

[22] **Pérez-Stable EJ, Nápoles-Springer A, Miramontes JM.** The effects of ethnicity and language on medical outcomes of patients with hypertension or diabetes. Med Care. 1997; 35:1212–9. 10.1097/00005650-199712000-00005 PMID: 9413309 . 9413309 10.1097/00005650-199712000-00005

[23] **Karam SL, Dendy J, Polu S, Blonde L.** Overview of Therapeutic Inertia in Diabetes: Prevalence, Causes, and Consequences. Diabetes Spectr. 2020; 33:8–15. 10.2337/ds19-0029 PMID: 32116448 . 32116448 10.2337/ds19-0029 PMC7026754

[24] **Lorenzati B, Zucco C, Miglietta S, Lamberti F, Bruno G.** Oral Hypoglycemic Drugs: Pathophysiological Basis of Their Mechanism of ActionOral Hypoglycemic Drugs: Pathophysiological Basis of Their Mechanism of Action. Pharmaceuticals (Basel). 2010; 3:3005–20. Epub 2010/09/15. 10.3390/ph3093005 PMID: 27713388 . 27713388 10.3390/ph3093005 PMC4034109

[25] **Galaviz KI, Narayan KMV, Lobelo F, Weber MB.** Lifestyle and the Prevention of Type 2 Diabetes: A Status Report. Am J Lifestyle Med. 2018; 12:4–20. Epub 2015/11/24. 10.1177/1559827615619159 PMID: 30202378 . 30202378 10.1177/1559827615619159 PMC6125024

[26] **Sami W, Ansari T, Butt NS, Hamid MRA.** Effect of diet on type 2 diabetes mellitus: A review. Int J Health Sci (Qassim). 2017; 11:65–71. 28539866 PMC5426415

[27] **Reddy PH.** Can Diabetes Be Controlled by Lifestyle Activities. Curr Res Diabetes Obes J. 2017; 1. PMC5792082 29399663

[28] **Chong E, Wang H, King-Shier KM, Quan H, Rabi DM, Khan NA.** Prescribing patterns and adherence to medication among South-Asian, Chinese and white people with type 2 diabetes mellitus: a population-based cohort study. Diabet Med. 2014; 31:1586–93. Epub 2014/09/15. 10.1111/dme.12559 PMID: 25131338 . 25131338 10.1111/dme.12559

[29] **Tocher TM, Larson E.** Quality of diabetes care for non-English-speaking patients. A comparative study. West J Med. 1998; 168:504–11. 9655991 PMC1305066

[30] **Choe AY, Thomson JE, Unaka NI, Wagner V, Durling M, Moeller D, et al.** Disparity in Nurse Discharge Communication for Hospitalized Families Based on English Proficiency. Hosp Pediatr. 2021; 11:245–53. Epub 2021/02/02. 10.1542/hpeds.2020-000745 PMID: 33531376 . 33531376 10.1542/hpeds.2020-000745 PMC7898234

[31] **Pérez-Stable EJ, El-Toukhy S.** Communicating with diverse patients: How patient and clinician factors affect disparities. Patient Educ Couns. 2018; 101:2186–94. Epub 2018/08/22. 10.1016/j.pec.2018.08.021 PMID: 30146407 . 30146407 10.1016/j.pec.2018.08.021 PMC6417094

[32] **Lee KC, Winickoff JP, Kim MK, Campbell EG, Betancourt JR, Park ER, et al.** Resident physicians' use of professional and nonprofessional interpreters: a national survey. JAMA. 2006; 296:1050–3. 10.1001/jama.296.9.1050 PMID: 16954482 . 16954482 10.1001/jama.296.9.1050

[33] **Diamond LC, Schenker Y, Curry L, Bradley EH, Fernandez A.** Getting by: underuse of interpreters by resident physicians. J Gen Intern Med. 2009; 24:256–62. Epub 2008/12/17. 10.1007/s11606-008-0875-7 PMID: 19089503 . 19089503 10.1007/s11606-008-0875-7 PMC2628994

[34] **Parker MM, Fernández A, Moffet HH, Grant RW, Torreblanca A, Karter AJ.** Association of Patient-Physician Language Concordance and Glycemic Control for Limited-English Proficiency Latinos With Type 2 Diabetes. JAMA Intern Med. 2017; 177:380–7. 10.1001/jamainternmed.2016.8648 PMID: 28114680 . 28114680 10.1001/jamainternmed.2016.8648 PMC5339062

[35] **Müller F, Holman H, Hummers E, Schröder D, Noack EM.** Multilingual competencies among ambulatory care providers in three German Federal States. BMC Prim Care. 2022; 23:315. Epub 2022/12/06. 10.1186/s12875-022-01926-1 PMID: 36474173 . 36474173 10.1186/s12875-022-01926-1 PMC9724318

[36] **LaVeist TA, Pierre G.** Integrating the 3Ds--social determinants, health disparities, and health-care workforce diversity. Public Health Rep. 2014; 129 Suppl 2:9–14. 10.1177/00333549141291S204 PMID: 24385659 . 24385659 10.1177/00333549141291S204 PMC3863706

[37] **Molina RL, Kasper J.** The power of language-concordant care: a call to action for medical schools. BMC Med Educ. 2019; 19:378. Epub 2019/11/06. 10.1186/s12909-019-1807-4. PMID: 31690300 . 31690300 10.1186/s12909-019-1807-4 PMC6833293

[38] **Bou Malham C, El Khatib S, Cestac P, Andrieu S, Rouch L, Salameh P.** Impact of pharmacist-led interventions on patient care in ambulatory care settings: A systematic review. Int J Clin Pract. 2021; 75:e14864. Epub 2021/09/20. 10.1111/ijcp.14864 PMID: 34523204 . 34523204 10.1111/ijcp.14864

[39] **Jourdan J-P, Muzard A, Goyer I, Ollivier Y, Oulkhouir Y, Henri P, et al.** Impact of pharmacist interventions on clinical outcome and cost avoidance in a university teaching hospital. Int J Clin Pharm. 2018; 40:1474–81. Epub 2018/10/26. 10.1007/s11096-018-0733-6 PMID: 30367375 . 30367375 10.1007/s11096-018-0733-6

[40] **Sarangarm P, London MS, Snowden SS, Dilworth TJ, Koselke LR, Sanchez CO, et al.** Impact of pharmacist discharge medication therapy counseling and disease state education: Pharmacist Assisting at Routine Medical Discharge (project PhARMD). Am J Med Qual. 2013; 28:292–300. Epub 2012/10/02. 10.1177/1062860612461169 PMID: 23033542 . 23033542 10.1177/1062860612461169

[41] **Bradshaw M, Tomany-Korman S, Flores G.** Language barriers to prescriptions for patients with limited English proficiency: a survey of pharmacies. Pediatrics. 2007; 120:e225-35. 10.1542/peds.2006-3151 PMID: 17671036 . 17671036 10.1542/peds.2006-3151

[42] **Johnson A, Liles L.** A Survey-Based Analysis of the Language of the Prescription Bottles and Instructions for the Medications of Limited English Proficiency Patients. Am J Med Qual. 2023; 38:113–4. Epub 2023/02/03. 10.1097/JMQ.0000000000000111 PMID: 36727576 . 36727576 10.1097/JMQ.0000000000000111

[43] **Masland MC, Kang SH, Ma Y.** Association between limited English proficiency and understanding prescription labels among five ethnic groups in California. Ethn Health. 2011; 16:125–44. 10.1080/13557858.2010.543950 PMID: 21491287 . 21491287 10.1080/13557858.2010.543950

[44] **Zargarzadeh AH, Law AV.** Access to multilingual prescription labels and verbal translation services in California. Res Social Adm Pharm. 2011; 7:338–46. Epub 2010/10/02. 10.1016/j.sapharm.2010.08.001 PMID: 21272528 . 21272528 10.1016/j.sapharm.2010.08.001

[45] **Tseng J, Halperin L, Ritholz MD, Hsu WC.** Perceptions and management of psychosocial factors affecting type 2 diabetes mellitus in Chinese Americans. J Diabetes Complications. 2013; 27:383–90. Epub 2013/03/29. 10.1016/j.jdiacomp.2013.01.001 PMID: 23545465 . 23545465 10.1016/j.jdiacomp.2013.01.001

[46] **Lu A-P, Jia H-W, Xiao C, Lu Q-P.** Theory of traditional Chinese medicine and therapeutic method of diseases. World J Gastroenterol. 2004; 10:1854–6. 10.3748/wjg.v10.i13.1854 PMID: 15222022 . 15222022 10.3748/wjg.v10.i13.1854 PMC4572216

[47] **Matos LC, Machado JP, Monteiro FJ, Greten HJ.** Understanding Traditional Chinese Medicine Therapeutics: An Overview of the Basics and Clinical Applications. Healthcare (Basel). 2021; 9. 10.3390/healthcare9030257. 10.3390/healthcare9030257 PMC8000828 33804485

[48] **Gordon NP, Iribarren C.** Health-related characteristics and preferred methods of receiving health education according to dominant language among Latinos aged 25 to 64 in a large Northern California health plan. BMC Public Health. 2008; 8:305. Epub 2008/09/09. 10.1186/1471-2458-8-305 PMID: 18782454 . 18782454 10.1186/1471-2458-8-305 PMC2556675

[49] **Tørris C, Nortvedt L.** Health literacy and self-care among adult immigrants with type 2 diabetes: a scoping review. BMC Public Health. 2024; 24:3248. Epub 2024/11/22. 10.1186/s12889-024-20749-6 PMID: 39578821 . 39578821 10.1186/s12889-024-20749-6 PMC11583541

[50] **Kershen AJ.** Food in the Migrant Experience. Routledge; 2002.

[51] **Satia-Abouta J, Patterson RE, Neuhouser ML, Elder J.** Dietary acculturation: applications to nutrition research and dietetics. J Am Diet Assoc. 2002; 102:1105–18. 10.1016/S0002-8223(02)90247-6 PMID: 12171455 . 12171455 10.1016/s0002-8223(02)90247-6

[52] **Himmelgreen D, Pérez-Escamilla R, Segura-Millán S, Peng Y-K, Gonzalez A, Singer M, et al.** Food Insecurity Among Low-Income Hispanics in Hartford, Connecticut: Implications for Public Health Policy. Human Organization. 2000; 59:334–42. 10.17730/humo.59.3.76557m317748l414.

[53] **Carrasquillo O, Carrasquillo AI, Shea S.** Health insurance coverage of immigrants living in the United States: differences by citizenship status and country of origin. Am J Public Health. 2000; 90:917–23. 10.2105/ajph.90.6.917 PMID: 10846509 . 10846509 10.2105/ajph.90.6.917 PMC1446276

[54] **Tarraf W, Jensen GA, Li Y, Toseef MU, Mahmoudi E, Gonzalez HM.** Changes in Insurance Coverage and Healthcare Use Among Immigrants and US-Born Adults Following the Affordable Care Act. J Racial Ethn Health Disparities. 2021; 8:363–74. Epub 2020/07/03. 10.1007/s40615-020-00790-y PMID: 32621099 . 32621099 10.1007/s40615-020-00790-y

[55] **Bowker SL, Mitchell CG, Majumdar SR, Toth EL, Johnson JA.** Lack of insurance coverage for testing supplies is associated with poorer glycemic control in patients with type 2 diabetes. CMAJ. 2004; 171:39–43. 10.1503/cmaj.1031830 PMID: 15238494 . 15238494 10.1503/cmaj.1031830 PMC437682

